# Editorial: Advances in understanding mucosal immunity in coronaviruses: from mechanisms to vaccines

**DOI:** 10.3389/fimmu.2025.1702286

**Published:** 2025-09-30

**Authors:** Farhat Afrin, Qibo Zhang, Wael Alturaiki, Waleed Mahallawi

**Affiliations:** ^1^ Centre for Interdisciplinary Sciences, JIS Institute of Advanced Studies and Research, JIS University, Kolkata, West Bengal, India; ^2^ Academic and Research Departments, Section of Immunology, School of Biosciences, University of Surrey, Surrey, United Kingdom; ^3^ Medical Laboratories Sciences Department, College of Applied Medical Science, Majmaah University, Al Majmaah, Saudi Arabia; ^4^ Clinical Laboratory Sciences Department, College of Applied Medical Sciences, Taibah University, Medina, Saudi Arabia

**Keywords:** mucosal immunity, coronaviruses, vaccines, respiratory viral coinfections, cross reactivity, immuno-epigenetic regulation, immune evasion, long-COVID

The emergence of severe acute respiratory syndrome coronavirus (SARS-CoV) in 2002, Middle East respiratory syndrome coronavirus (MERS-CoV) in 2012, and SARS-CoV-2 in 2019 has highlighted the significant threat coronaviruses pose to global health. While systemic immunity has traditionally been the main focus of vaccine development, it is now increasingly clear that mucosal immunity plays a crucial role in determining the outcome of coronavirus infections. The mucosa of the respiratory tract serves as the primary contact point with the virus, and immune responses initiated at this interface can prevent viral entry, limit replication, and influence the progression of systemic disease. This Research Topic brings together five contributions that enhance our understanding of the mechanisms underlying mucosal immunity in coronaviruses and explore innovative strategies for vaccine development. Collectively, they emphasize key aspects of host-pathogen interactions, ranging from immuno-epigenetic regulation to sex-biased vaccine responses and methodological advancements in measuring mucosal antibodies.

Gupta et al. provide an in-depth review of the epigenetic mechanisms shaping host responses to SARS-CoV-2 infection. Beyond the viral genome and protein repertoire, the authors emphasize the role of host-directed regulatory processes such as DNA methylation, histone modifications, chromatin remodeling, and non-coding RNAs that modulate antiviral gene expression and influence susceptibility to COVID-19. These reversible changes may contribute to viral persistence, immune evasion, and heterogeneity in disease severity. Importantly, Gupta et al. highlight potential therapeutic opportunities within immuno-epigenetic pathways, pointing towards host-directed therapies that could complement antivirals. This perspective is consistent with recent studies showing that epigenetic signatures can stratify COVID-19 outcomes and predict long-COVID risk (1).

Shrwani et al. have reviewed the mucosal immune responses in the upper and lower respiratory tracts during MERS-CoV infection. With a case fatality rate of approximately 36%, MERS-CoV represents one of the most lethal human coronaviruses. The review integrates evidence from 152 studies, detailing the interplay of innate immune cells such as macrophages, dendritic cells, and NK cells, with adaptive responses mediated by CD4^+^ and CD8^+^ T cells and B cells. Importantly, the authors draw attention to the role of nasopharynx-associated lymphoid tissue (NALT) and bronchus-associated lymphoid tissue (BALT) as local immunological niches that could be harnessed by vaccines. Yet, as they note, the scarcity of human tissue studies limits our understanding of mucosal immunopathogenesis in MERS-CoV, reinforcing the need for translational models.

Alqahtani provides a comprehensive review of mucosal immunity in COVID-19, highlighting its pivotal role in both early viral restriction and long-term immune protection. While most clinical research has focused on systemic antibody and T-cell responses in peripheral blood, the review underscores that secretory IgA, tissue-resident T cells, and local cytokine responses in the respiratory tract are critical determinants of SARS-CoV-2 clearance. The review also emphasizes how coronaviruses deploy immune evasion strategies at mucosal surfaces, blunting detection and neutralization. An important forward-looking aspect of this work is the discussion of mucosal vaccines. Clinical trials of intranasal and oral vaccines have shown promise in generating sterilizing immunity, reducing viral shedding, and enhancing cross-variant protection (4, 5). By synthesizing evidence on mucosal biomarkers and vaccine responses, Alqahtani’s review makes a compelling case for integrating mucosal endpoints into COVID-19 vaccine development and surveillance.

Moving from reviews to experimental studies, Li et al. explore the sex-specific immunogenicity of a recombinant intranasal subunit vaccine in mice. The study demonstrates that intranasal vaccination elicits robust systemic IgG, nasal and bronchoalveolar IgA, and lung T-cell responses, but with clear sex differences in magnitude and quality. Notably, monocytes, alveolar macrophages, and CD103^+^ dendritic cells correlated with enhanced mucosal responses. These findings extend prior knowledge that sex differences influence systemic vaccine responses (2), highlighting that similar biases exist in mucosal immunity. This work has direct implications for vaccine design and evaluation. Sex as a biological variable must be incorporated into preclinical and clinical trials, both to optimize protective efficacy and to avoid unrecognized disparities in vaccine performance.

Bladh et al. address a methodological gap in mucosal vaccine research by comparing SARS-CoV-2 spike-specific IgA and IgG across nasal secretions, saliva, and serum in a large cohort of healthcare workers. Their findings reveal that spike-specific IgA levels are markedly higher in nasal secretions than saliva, with stronger correlations between nasal IgA and secretory IgA (sIgA). Importantly, sIgA showed superior cross-binding capacity to SARS-CoV-2 variants compared to serum IgG or monomeric IgA. This study emphasizes two key points. First, nasal secretions are a superior compartment for assessing mucosal antibody responses. Second, sIgA plays a critical role in cross-variant protection, suggesting that mucosal vaccines should be evaluated not just for systemic neutralizing antibodies but also for their ability to elicit durable sIgA responses. These insights are consistent with recent reports demonstrating that mucosal IgA responses correlate with reduced viral transmission in vaccinated populations (3).

Together, the five contributions to this Research Topic converge on three overarching themes. First, mucosal immunity is central to coronavirus defense, shaping the early course of infection and influencing systemic outcomes. Future vaccine strategies must move beyond systemic neutralization and incorporate mucosal endpoints, including sIgA and tissue-resident T cells. Second, epigenetic and host-directed mechanisms are emerging as critical regulators of mucosal immunity. Integrating epigenomic profiling with immunological studies may reveal biomarkers of protection and susceptibility, as well as novel therapeutic targets. Third, methodological standardization and inclusion of biological variables such as sex are essential. Harmonized protocols for sample collection, antibody quantification, and mucosal biomarker assessment will enable more reliable comparisons across trials and populations. In the broader context, the lessons from SARS-CoV-2 and MERS-CoV extend beyond coronaviruses. Respiratory pathogens such as influenza, RSV, and emerging zoonotic viruses share mucosal entry routes and immune challenges. Advances in mucosal immunology and vaccine delivery platforms will therefore have cross-cutting benefits for pandemic preparedness.

To conclude, this Research Topic underscores the dynamic advances in understanding mucosal immunity in coronaviruses, from fundamental mechanisms to translational applications. By integrating insights from epigenetic regulation, MERS-CoV immunopathogenesis, COVID-19 mucosal responses, sex-biased vaccine effects, and antibody quantification methods, the contributions collectively provide a roadmap for the next generation of vaccines and therapeutics. As the field moves forward, prioritizing mucosal immunity while embracing interdisciplinary approaches that span epigenetics, immunology, and vaccinology will be pivotal for controlling coronaviruses and safeguarding global health.
